# Aortic valve replacement in patients aged 50 to 69 years: Analysis using Korean National Big Data

**DOI:** 10.1111/jocs.16908

**Published:** 2022-09-02

**Authors:** Min‐Seok Kim, Hae Rim Kim, Seung Hyun Lee, Sak Lee, Hyun‐Chel Joo

**Affiliations:** ^1^ Department of Thoracic and Cardiovascular Surgery, Cardiovascular Center, Myongji Hospital Hanyang University College of Medicine Seoul Korea; ^2^ Department of Statistics, College of Natural Science University of Seoul Seoul Korea; ^3^ Division of Cardiovascular Surgery, Severance Cardiovascular Hospital Yonsei University College of Medicine Seoul Korea

**Keywords:** valve repair/replacement

## Abstract

**Background:**

The aim of this study was to compare the clinical outcomes and long‐term survival in patients who underwent isolated aortic valve replacement (AVR) with mechanical versus bioprosthetic valves.

**Methods:**

Patients aged 50–69 years who had undergone AVR from 2002 to 2018 were identified and their characteristics were collected from Korean National Health Information Database formed by the National Health Insurance Service, Republic of Korea. Of the 5792 patients, 1060 patients were excluded due to missing values on characteristics. Of the 4732 study patients, 1945 patients (41.1%) had received bioprosthetic valves (Group B) and 2787 patients (58.9%) had received mechanical valves (Group M). A propensity score‐matched analysis was performed to match 1429 patients in each group. Data on mortality, cardiac mortality, reoperations, cerebrovascular accidents, and bleeding complications were obtained.

**Results:**

The overall survival rates at 5 and 10 years postoperatively were 87.8% and 75.2% in the matched Group B and 91.2% and 76.7% in the matched Group M, respectively (*p* = .140). Freedom from cardiac death rates at postoperative 5 and 10 years were 95.6% and 92.4% in the matched Group B and 96.0% and 92.1% in the matched Group M, respectively (*p* = .540). The cumulative incidence of reoperation was higher in the matched Group B than in the matched Group M (*p* = .007), and the cumulative incidence of major bleeding was higher in the matched Group M than in the matched Group B (*p* = .039).

**Conclusion:**

In patients aged 50–69 years who underwent isolated AVR, the patients who received bioprosthetic valves showed similar cardiac mortality‐free survival and long‐term survival rates to the patients who received mechanical valves.

## 1 INTRODUCTION

Aortic valve replacement ([AV]R) is the standard treatment in patients with severe AV disease, and has shown improvement of survival, quality of life, and left ventricular (LV) function.^1^, ^2^, ^3^ The choice of a valve prosthesis used in AVR is a complex and individualized decision which is based on valve‐related factors such as valve durability, hemodynamics and reintervention risks, and patient factors, such as lifestyle, preferences, life expectancy, and adherence to medication.^4^, ^5^ Of the various factors in making decision of the type of a valve prosthesis, patient's age is one of the most important factors. Current AVR guidelines from the American Heart Association/American College of Cardiology states that it is reasonable to choose mechanical prosthesis in patients <50 years of age and biological prosthesis in patients >65 years of age. Either type of prosthesis is considered reasonable in patients 50–65 years of age by individualizing the patient's status and condition.^6^ As for the European Society of Cardiology/European Association for Cardio‐Thoracic Surgery AVR guidelines, mechanical prosthesis should be considered in patients <60 years of age and biological prosthesis in patients >65 years of age. In patients 60–65 years of age, both types of prosthesis are considered acceptable.^7^ Previous studies have compared the long‐term survival and clinical outcomes after AVR with mechanical versus bioprosthetic valves in these “middle‐aged” patients.^8^, ^9^, ^10^, ^11^, ^12^, ^13^, ^14^ Conflicting results, however, have been reported on clinical outcomes and long‐term survival.

The aim of the present study was to compare the clinical outcomes and long‐term survival in patients who underwent isolated AVR with mechanical versus bioprosthetic valves by analyzing the Korean national big data.

## 2 PATIENTS AND METHODS

### 2.1 Study design

This nationwide, retrospective cohort study protocol was reviewed by the Institutional Review Board and approved as a minimal‐risk retrospective study (Approval Date: August 3rd, 2018; Approval Number: 4‐2018‐0588) that did not require individual consent based on the institutional guidelines for waiving consent.

**Figure 1:**
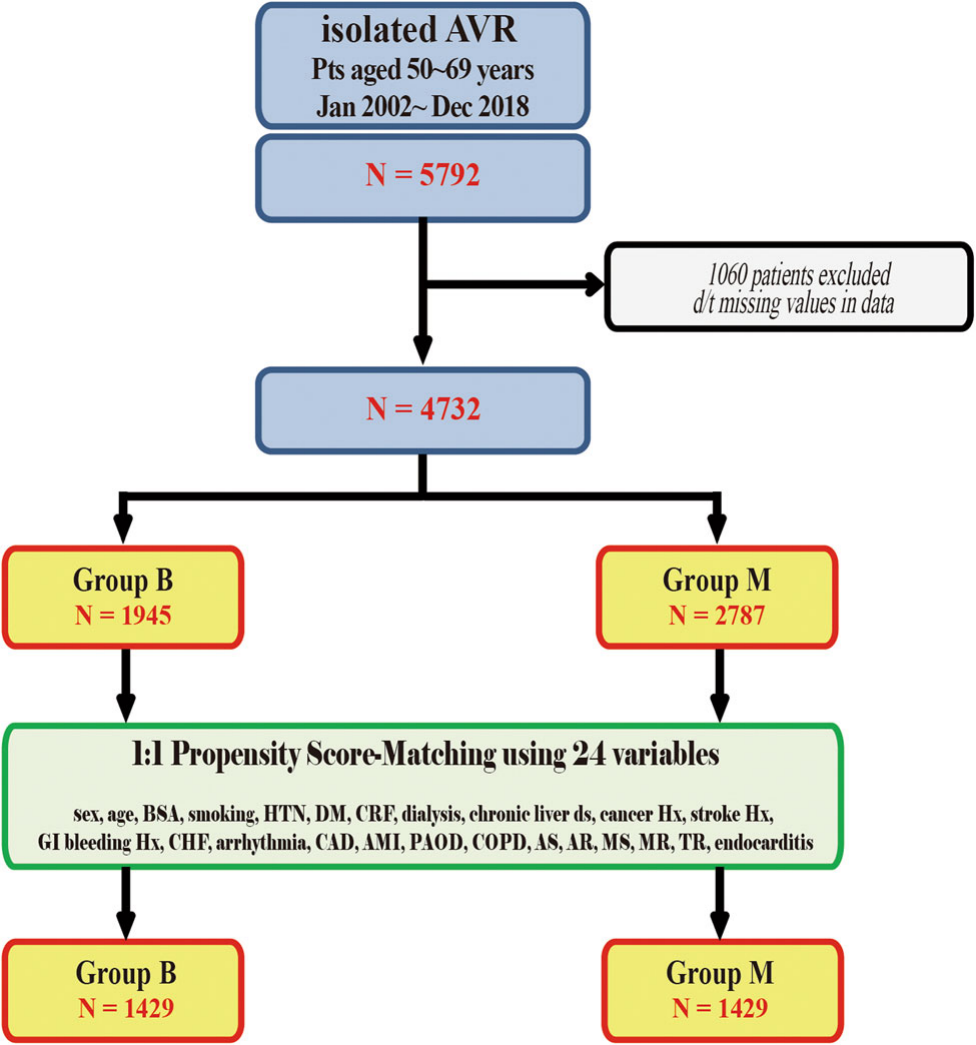
Summary flow diagram of patients (Group B, patients who underwent AVR with biological prosthesis; Group M, patients who underwent AVR with mechanical prosthesis). AMI, acute myocardial infarction; AR, aortic regurgitation; AS, aortic stenosis; AVR, aortic valve replacement; BSA, body surface area; CAD, coronary artery disease; CHF, congestive heart failure; COPD, chronic obstructive pulmonary disease; CRF, chronic renal failure; DM, diabetes mellitus; HTN, hypertension; MR, mitral regurgitation; MS, mitral stenosis; PAOD, peripheral arterial occlusive disease; TR, tricuspid regurgitation

Data were collected from National Health Information Database provided by National Health Insurance Service of Republic of Korea, which is the mandatory health insurance scheme covering the whole Korean population. A total of 5792 patients aged 50–69 years who underwent first‐time isolated AVR between January 1st, 2002 and December 31th, 2018 in Republic of Korea were included. Patients who underwent other concomitant procedures with AVR (such as mitral valve replacement or repair, tricuspid valve replacement or repair, coronary artery bypass grafting, surgery on ascending aorta or aortic arch, etc.) and patient who underwent redo‐AVR were excluded. Total of 5792 patients (bioprosthesis group vs. mechanical prosthesis group; 2381 vs. 3411 patients) were initially included in the present study. Preoperative patient characteristics, information on the valves used for AVR (bioprosthetic vs. mechanical valves), and postoperative clinical data (including death, cerebrovascular events, and major bleeding) were collected. After exclusion of 1060 patients who had missing values of patient data, 4732 patients were finally included in this study; 1945 patients (41.1%) who had received bioprosthetic valves (Group B) and 2787 patients (58.9%) who had received mechanical valves (Group M). Propensity score‐matched analysis using 24 variables was performed to adjust differences in preoperative characteristics, and 1429 patients in each group were extracted by 1:1 matching. Before matching, Group M patients were younger, had more smokers, larger BSA, more male gender, less chronic obstructive pulmonary disease, less diabetes mellitus, less cancer, and more aortic regurgitation. After matching, however, there were no differences in demographic data between the matched groups. After matching, all covariates were well balanced between the groups with standardized mean difference ≤10% (Figure 1 and Table 1). Data on reoperations (redo‐AVR), cerebrovascular accidents, and major bleeding complications during the follow‐up were collected by using National Health Information Database. Thirty‐day mortalities was defined as any death within 30 days of procedure, including deaths after hospital discharge. Cerebrovascular accidents include stroke and transient ischemic attack, and major bleeding complications include brain hemorrhage and gastrointestinal hemorrhage. To obtain information on patients' survival and cause of death, we have additionally obtained data for the vital statistics and death from cardiovascular disease from death certificates available at Statistics Korea, a central organization for statistics under Ministry of Strategy and Finance.

**Table 1:** Comparison of Groups B and M before and after propensity score‐matching

	All study patients			Propensity score‐matched patients			
	Group B (*N* = 2381)	Group M (*N* = 3411)	*p*	Group B (*N* = 1429)	Group M (*N* = 1429)	*p*	SMD
Age (year)	64.9 ± 4.0	59.2 ± 5.1	<.001	63.1 ± 4.2	62.9 ± 4.3	.250	0.043
Female	914 (38.4%)	1190 (34.9%)	.007	522 (36.5%)	529 (37.0%)	.816	0.010
BSA (m^2^)	1.67 ± 0.16	1.70 ± 0.16	<.001	1.69 ± 0.15	1.69 ± 0.16	.870	0.006
Smoking			<.001			.395	0.065
Nonsmoker	1591 (66.8%)	2100 (61.6%)		919 (64.3%)	895 (62.7%)		
Ex‐smoker	483 (20.3%)	834 (24.5%)		315 (22.0%)	340 (23.8%)		
Current smoker	307 (12.9%)	477 (14.0%)		195 (13.6%)	194 (13.6%)		
Hypertension	779 (32.7%)	1042 (30.5%)	.206	467 (32.7%)	467 (32.7%)	.361	0.053
Diabetes mellitus	634 (26.6%)	719 (21.1%)	<.001	359 (25.1%)	357 (25.0%)	.360	0.054
Chronic renal failure	125 (5.2%)	157 (4.6%)	.322	69 (4.8%)	65 (4.5%)	.939	0.013
Dialysis	32 (1.3%)	44 (1.3%)	.566	18 (1.3%)	17 (1.2%)	.986	0.006
Chronic liver disease	72 (3.0%)	77 (2.3%)	.192	37 (2.6%)	40 (2.8%)	.386	0.052
History of cancer	132 (5.5%)	131 (3.8%)	.009	76 (5.3%)	71 (5.0%)	.339	0.055
History of stroke	56 (2.4%)	61 (1.8%)	.160	29 (2.0%)	28 (2.0%)	>.999	0.009
History of gastrointestinal bleeding	110 (4.6%)	142 (4.2%)	.439	63 (4.4%)	66 (4.6%)	.857	0.018
Congestive heart failure	216 (9.1%)	280 (8.2%)	.318	121 (8.5%)	122 (8.5%)	.998	0.003
Arrhythmia	111 (4.7%)	173 (5.1%)	.427	73 (5.1%)	74 (5.2%)	.996	0.003
Coronary artery disease	108 (4.5%)	136 (4.0%)	.356	64 (4.5%)	62 (4.3%)	0.983	0.007
Acute myocardial infarction	3 (0.1%)	1 (0.0%)	.223	0 (0.0%)	0 (0.0%)	>0.999	<0.001
Peripheral vascular obstructive disease	5 (0.2%)	7 (0.2%)	.572	5 (0.3%)	2 (0.1%)	.525	0.042
Chronic obstructive pulmonary disease	128 (5.4%)	119 (3.5%)	.001	75 (5.2%)	73 (5.1%)	.986	0.006
Aortic stenosis	2052 (86.2%)	2658 (77.9%)	<.001	1203 (84.2%)	1224 (85.7%)	.296	0.059
Aortic regurgitation	768 (32.3%)	1542 (45.2%)	<.001	540 (37.8%)	558 (39.0%)	.513	0.041
Mitral stenosis	20 (0.8%)	29 (0.9%)	.571	13 (0.9%)	13 (0.9%)	>.999	<0.001
Mitral regurgitation	51 (2.1%)	80 (2.3%)	.492	33 (2.3%)	29 (2.0%)	.876	0.019
Tricuspid regurgitation	0 (0.0%)	1 (0.0%)	.403	0 (0.0%)	0 (0.0%)	>.999	<0.001
Endocarditis	76 (3.2%)	118 (3.5%)	.478	44 (3.1%)	45 (3.1%)	.994	0.004

The clinical follow‐up were closed on July 31, 2020. The follow‐up data were complete in 100% of patients, with a median follow‐up duration of 60.5 months ([47.0, 90.0] months).

### 2.2 Statistical analysis

Statistical analysis was performed with R software, version 3.6.0 (R Foundation for Statistical Computing, Vienna, Austria). Continuous data were expressed as the mean ± standard deviation for normally distributed variables or as medians (interquartile range) for non‐normally distributed variables according to the Shapiro–Wilk test, and categoric data were expressed as count (percentage). Comparison between continuous variables were made using the Student *t* test, and categoric variables were made using a Chi‐squared test. For propensity score‐matching, 24 variables, including sex, age, body surface area, smoking status, hypertension, diabetes mellitus, chronic renal failure, dialysis, chronic liver disease, history of cancer, history of stroke, history of gastrointestinal bleeding, congestive heart failure, arrhythmia, coronary artery disease, acute myocardial infarction, peripheral arterial obstructive disease, chronic obstructive pulmonary disease, aortic stenosis, aortic regurgitation, mitral stenosis, mitral regurgitation, tricuspid regurgitation, and endocarditis were used. A propensity score‐matching analysis was performed using R software (MatchIt package), and nearest neighbor matching was used to match the groups in 1:1 manner. After propensity score‐matching, overall survival and freedom from cardiac death were analyzed using Kaplan–Meier Survival curves and cumulative incidence of reoperation, stroke and major bleeding complications were analyzed using cumulative incidence function with death as a competing risk. Comparison between the matched groups were performed using the stratified log‐rank test for overall survival and freedom from cardiac death, and robust standard errors in the Fine‐Gray model for cumulative incidence functions. The Cox proportional hazard model was used to identify risk factors that affect the mortality and cardiac mortality. Variables that achieved *p* < .05 in the univariable analysis were entered into the multivariable analysis.

## 3 RESULTS

### 3.1 Trend in selection of the prostheses

**Figure 2:**
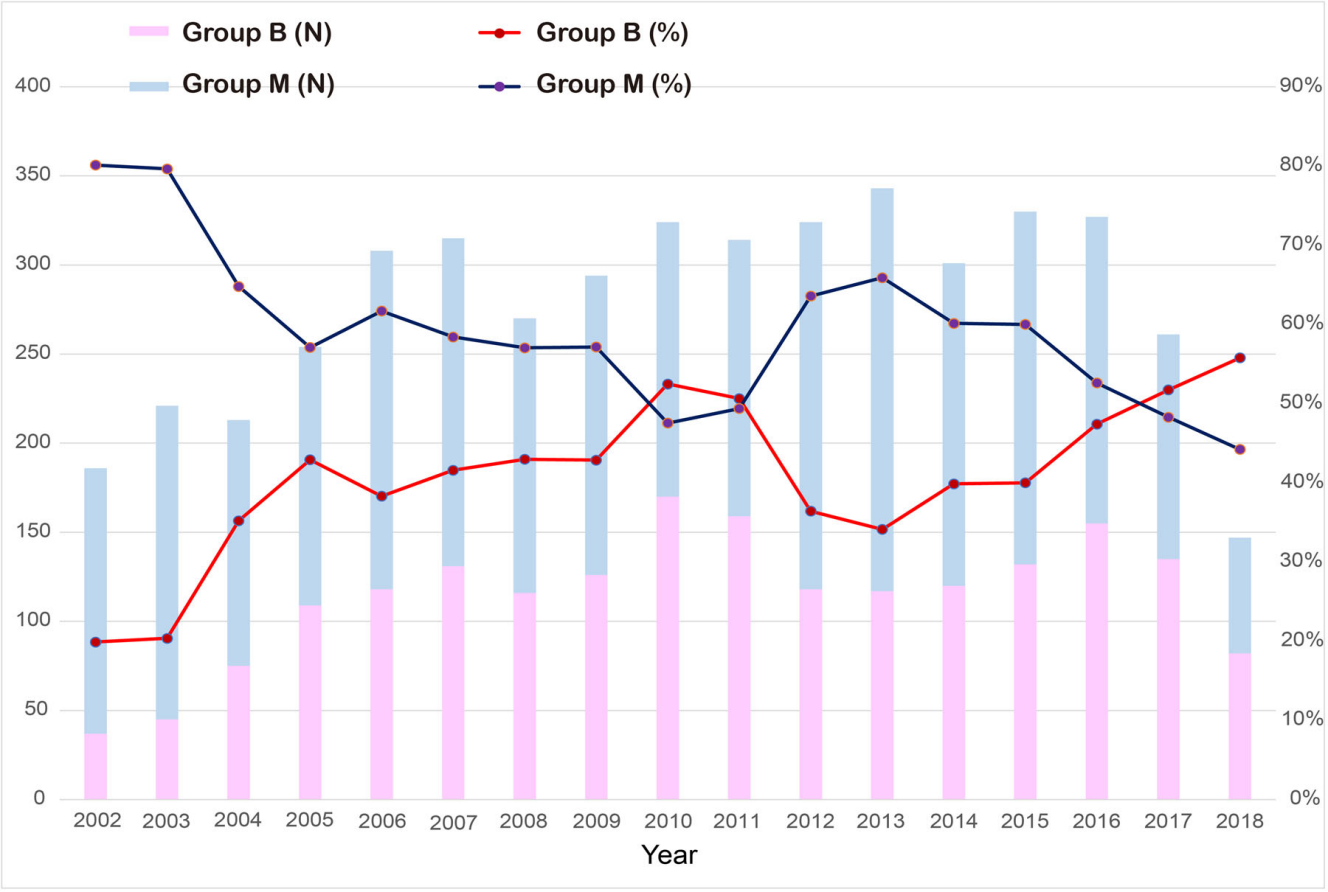
Aortic valve replacement per year in patients aged 50–69 years in Republic of Korea. Changes in number and ratio of patients who underwent aortic valve replacement with biological (Group B) or mechanical (Group M) prosthesis

In the 2002, over 80% of isolated AVR was performed using mechanical prostheses in patients aged 50–69 years old. However, the difference between the bioprosthetic and mechanical valves decreased after 2005. In the year of 2017 and 2018, the patients who received AVR using mechanical valves outnumbered the patients who received AVR using bioprosthetic valves (Figure 2).

### 3.2 Overall survival and freedom from cardiac deaths

**Figure 3:**
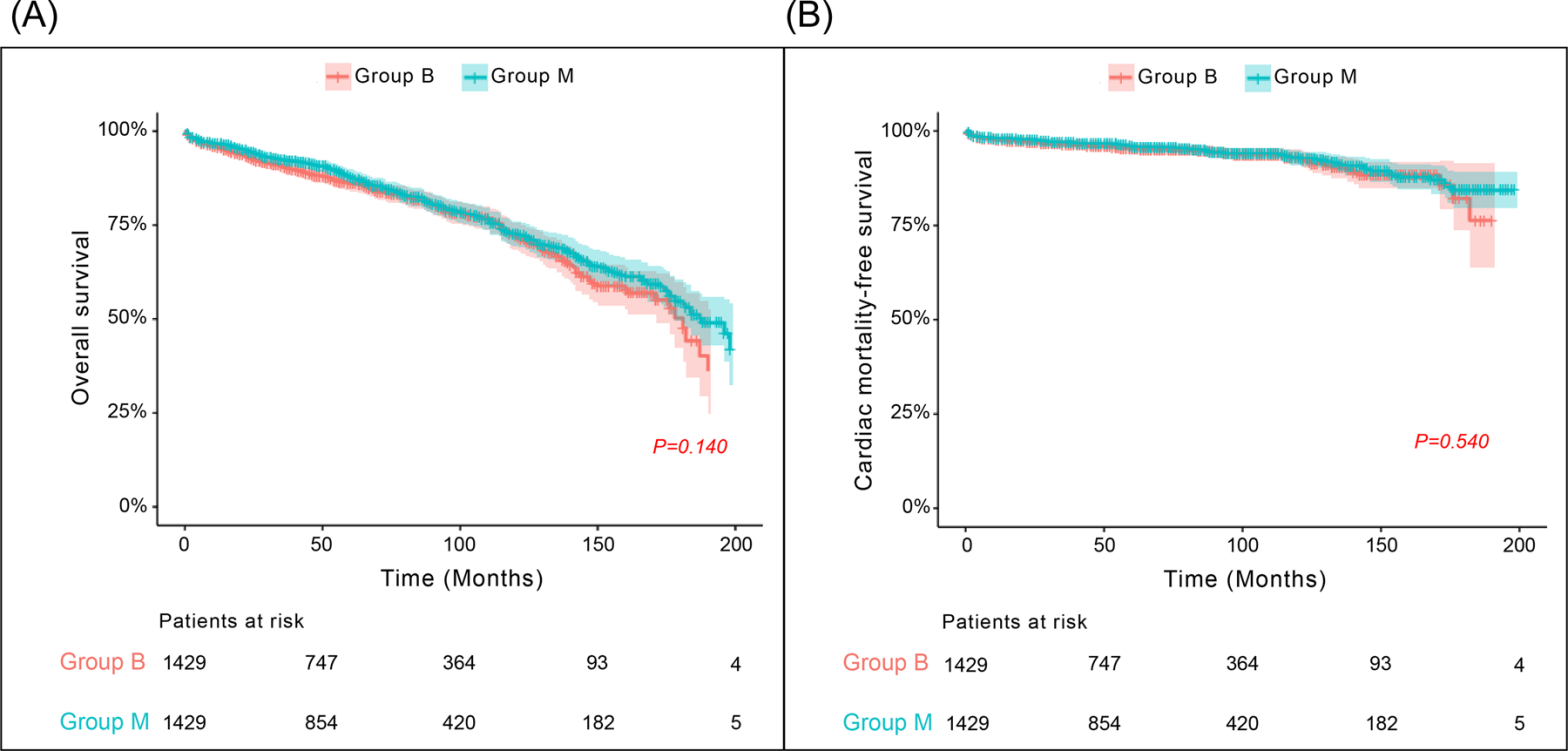
Comparison of (A) overall survival and (B) cardiac mortality‐free survival between the matched Groups B and M. All *p* values were obtained using stratified log‐rank tests. Group B, patients who underwent AVR with biological prosthesis; Group M, patients who underwent AVR with mechanical prosthesis. AVR, aortic valve replacement

During median follow‐up duration of 60.5 (47.0, 90.0) months, all‐cause overall mortalities occurred in 531 patients (263 in the matched group B and 268 in the matched Group M), including 155 cardiac deaths (88 in the matched Group B and 67 in the matched Group M). Thirty‐day mortalities occurred in 27 patients (12 in the matched Group B and 15 in the matched Group M). The overall survival rates at 5 and 10 years postoperatively were 87.8% and 75.2% in the matched Group B and 91.2% and 76.7% in the matched Group M, respectively (*p* = .140). Freedom from cardiac death rates at 5 and 10 years postoperatively were 95.6% and 92.4% in the matched Group B and 96.0% and 92.1% in the matched Group M, respectively (*p* = .540) (Figure 3).

### 3.3 Cumulative incidence of reoperation, stroke, and major bleeding

**Figure 4:**
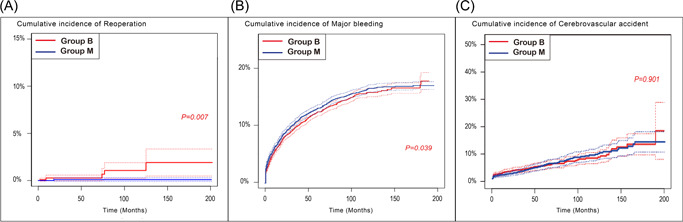
Comparison of cumulative incidence of (A) reoperation, (B) major bleeding, and (C) cerebrovascular accident between the matched Groups B and M, using competing risk analysis considering death as a competing risk. Group B, patients who underwent AVR with biological prosthesis; Group M, patients who underwent AVR with mechanical prosthesis. AVR, aortic valve replacement

There were 15 reoperations in all population during the study period. After the propensity score matching, there were 6 reoperations (5 in the matched Group B and 1 in the matched Group M) during the study period. When the cumulative incidence of reoperation was analyzed using competing risk analysis considering death as a competing risk, it was higher in the matched Group B than in the matched Group M (*p* = .007). The cumulative incidence of major bleeding was higher in the matched Group M than in the matched Group B (*p* = .039). No statistically significant differences were found in comparison of the cumulative incidence of stroke between the matched groups (*p* = .901) (Figure 4).

### 3.4 Predictors of all‐cause mortality and cardiac mortality

Univariable analysis by Cox proportional hazard model revealed age (hazard ratio [HR]: 0.72; 95% confidence interval [CI]: 0.58–0.89; *p* = .002), smoking (HR: 1.96; 95% CI: 1.17–3.29; *p* = .011), diabetes mellitus (HR: 1.67; 95% CI: 1.08–2.59; *p* = .022), chronic renal failure (HR: 13.28; 95% CI: 4.10–42.97; *p* < .001), congestive heart failure (HR: 2.58; 95% CI: 1.39–4.78; *p* = .003), and history of stroke (HR: 3.19; 95% CI: 2.01–5.05; *p* < .001) to be significant predictors of all‐cause mortality. When multivariable analysis was performed, age, smoking, diabetes mellitus, chronic renal failure, and history of stroke were statistically significant (Table 2A). Univariable analysis by Cox proportional hazard model revealed chronic renal failure (HR: 15.56; 95% CI: 2.05–117.78; *p* = .008) and history of stroke (HR: 6.69; 95% CI: 2.27–19.77; *p* < .001) to be significant predictors of all‐cause mortality, and these variables were also statistically significant predictors by multivariable analysis (Table 2B).

**Table 2A:** Predictors of all‐cause mortality

Variables	Univariable analysis		Multivariable analysis	
	HR (95% CI)	*p* value	HR (95% CI)	*p* value
Mechanical prosthesis	0.84 (0.67–1.06)	.136		
Age	0.72 (0.58–0.89)	.002	1.20 (1.08–1.31)	<.001
Male sex	1.33 (0.95–1.88)	.101		
Smoking	1.96 (1.17–3.29)	.011	2.25 (2.12–2.38)	<.001
BSA	1.45 (0.47–4.40)	.517		
Hypertension	1.03 (0.71–1.49)	.895		
Diabetes Mellitus	1.67 (1.08–2.59)	.022	1.30 (1.19–1.41)	.020
Coronary artery disease	1.05 (0.52–2.12)	.895		
Chronic renal failure	13.28 (4.10–42.97)	<.001	3.90 (3.75–4.06)	<.001
Dialysis	2.08 (0.72–4.84)	.996		
Atrial fibrillation or flutter	0.92 (0.49–1.74)	.805		
Congestive heart failure	2.58 (1.39–4.78)	.003	1.06 (0.993–1.19)	.660
Peripheral arterial obstructive disease	2.00 (0.18–22.06)	.571		
Chronic obstructive pulmonary disease	1.10 (0.60–2.01)	.766		
Cancer	1.14 (0.55–2.38)	.721		
History of stroke	3.19 (2.01–5.05)	<.001	1.93 (1.81–2.04)	<.001
Chronic liver disease	1.06 (0.31–3.56)	.928		
Aortic stenosis	1.18 (0.80‐1.74)	.408		
Aortic regurgitation	0.80 (0.48–1.32)	.387		
Mitral stenosis	0.67 (0.11–3.99)	.657		
Mitral regurgitation	0.83 (0.25–2.73)	.763		
Endocarditis	1.32 (0.58–2.98)	.511		

Abbreviations: CI, confidence interval; HR, hazard ratio.

**Table 2B:** Predictors of cardiac mortality

Variables	Univariable analysis		Multivariable analysis	
	HR (95% CI)	*p* value	HR (95% CI)	*p* value
Mechanical prosthesis	0.88 (0.59–1.32)	.536		
Age	0.72 (0.49–1.06)	.100		
Male sex	1.56 (0.83–2.93)	.163		
Smoking	2.67 (0.94–7.63)	.066		
BSA	0.42 (0.06–2.99)	.384		
Hypertension	1.14 (0.59–2.20)	.693		
Diabetes Mellitus	1.70 (0.71–4.06)	.233		
Coronary artery disease	1.00 (0.29–3.45)	>.999		
Chronic renal failure	15.56 (2.05–117.78)	.008	5.13 (4.88–5.38)	<.001
Dialysis	2.06 (0.58–4.10)	.997		
Atrial fibrillation or flutter	0.83 (0.25–2.73)	.763		
Congestive heart failure	2.17 (0.67–7.09)	.198		
Peripheral arterial obstructive disease	1.80 (0.60–5.37)	.292		
Chronic obstructive pulmonary disease	0.82 (0.27–2.46)	.723		
Cancer	0.33 (0.04–2.97)	.321		
History of stroke	6.69 (2.27–19.77)	<.001	2.25 (1.89–2.62)	.025
Chronic liver disease	1.05 (0.34–2.56)	.821		
Aortic stenosis	1.51 (0.61–3.74)	.377		
Aortic regurgitation	0.89 (0.37–2.09)	.781		
Mitral stenosis	1.00 (0.06–15.99)	>.999		
Mitral regurgitation	3.00 (0.31–28.84)	.341		
Endocarditis	1.24 (0.27–5.61)	.781		

Abbreviations: CI, confidence interval; HR, hazard ratio.

## 4 COMMENT

This nationwide, propensity score‐matched study compared long‐term outcomes in patients aged 50–69 years who received AVR using mechanical versus bioprosthetic valves. The present study demonstrated two main findings. First, there were no statistically significant differences in freedom from cardiac death and long‐term survival rates up to 15 years after the surgery between the patients aged 50–69 years who received bioprosthetic valves and who received mechanical valves. Second, patients who received bioprosthetic valves showed lower bleeding event rates than in patients who received mechanical valves.

After performing analyses with propensity score‐matched 2304 patients aged 50–70 years old, who underwent AVR with mechanical versus bioprosthetic valves in Finland. Kytoe et al.^15^ have concluded that the AVR with mechanical valves were associated with lower mortality compared with those with bioprosthetic valves. Several studies also have revealed mechanical valves to be superior to bioprosthetic valves in terms of survival in these aged group of patients.^5^, ^9^, ^13^, ^14^, ^16^ Other studies, however, have failed to demonstrate superiority in terms of survival in the patients who underwent AVR with mechanical valves compared to those with bioprosthetic valves.^8^, ^10^, ^11^, ^12^, ^17^, ^18^, ^19^

In the present study, there were no differences between the two groups in survival as well as cardiac mortality‐free survival. The results of the study are meaningful since relatively large number of 5792 nationwide patients aged 50–69 years who underwent isolated AVR were initially included in the present study, and propensity score‐matching was performed to decrease the possible source of bias and hence increase the statistical power of analyses. Similarly, by including relatively large number of 4253 patients and performing propensity score‐matching, Chiang et al have analyzed 1001 matched patients in each group and have concluded that no difference in survival rate was observed with bioprosthetic compared with mechanical valves.^11^

No significant differences were found in overall survival as well as cardiac mortality‐free survival between patients who underwent AVR with biological prosthesis and mechanical prosthesis in the present study. The result of the study may have more statistical significance since the study was conducted by using the National big data, and propensity score matching was performed.

Necessity of reoperation and risk of bleeding are major strengths and weaknesses of the two different types of prosthetic valves. Other studies have revealed mechanical valves to be superior than biological prostheses in terms of reoperation^8^, ^9^, ^10^, ^11^, ^12^, ^13^, ^15^, ^16^, ^18^, ^20^ and biological prostheses to be superior in terms of bleeding.^9^, ^11^, ^13^, ^16^, ^20^ In the present study, similarly, reoperation was more frequent in patients who underwent AVR with biological prosthesis, and major bleeding event more frequent in patients who underwent AVR with mechanical prosthesis. However, we are careful to draw a conclusion on reoperations because the absolute events were too small and the follow‐up period of this study was relatively short compared to the currently known longevity of bioprosthetic valves. Further information after 15 years of follow‐up is warranted.

Although the necessity of reoperation and risk of bleeding are the major points that are regarded in selecting the type of prosthesis used in AVR, no significant difference in the survival makes the decision more freely or patient‐tailored in selecting the type of prosthesis in patients aged 50–69 years who is undergoing isolated AVR.

### 4.1 Limitations of the study

The present study has limitations that must be recognized. First, the data were collected from National Health Information Database and there were limitations in collecting patients' characteristics and in‐hospital outcome data including preoperative LV function, EuroSCORE or STS score. Second, 1060 patients who had missing values on parameters were excluded from the study. Third, the follow‐up period was relatively short. Since the longevity of bioprosthetic valves is known to be about 15 years, further information after 15 years of follow‐up is warranted. Because of the relatively short follow‐up period, conclusions on certain outcomes such as reoperations should be drawn carefully, since the absolute events were too small during the study period.

## 5 CONCLUSIONS

In patients aged 50–69 years who underwent isolated AVR with biological prosthesis showed similar long‐term survival as well as cardiac mortality‐free survival rates compared to the patients who received mechanical prosthesis. The type of prosthesis used in isolated AVR should be decided more liberally in this aged group of patients, regarding other patient‐related factors.

## CONFLICTS OF INTEREST

The authors declare no conflicts of interest.
